# Lipopolysaccharide from the commensal microbiota of the breast enhances cancer growth: role of S100A7 and TLR4

**DOI:** 10.1002/1878-0261.12975

**Published:** 2021-11-16

**Authors:** Tasha Wilkie, Ajeet K. Verma, Helong Zhao, Manish Charan, Dinesh K. Ahirwar, Sashi Kant, Vijay Pancholi, Sanjay Mishra, Ramesh K. Ganju

**Affiliations:** ^1^ Department of Pathology The Ohio State University Wexner Medical Center Columbus OH USA

**Keywords:** breast cancer, LPS, microbiota, RAGE, S100A7, TLR4

## Abstract

The role of commensal bacterial microbiota in the pathogenesis of human malignancies has been a research field of incomparable progress in recent years. Although breast tissue is commonly assumed to be sterile, recent studies suggest that human breast tissue may contain a bacterial microbiota. In this study, we used an immune‐competent orthotopic breast cancer mouse model to explore the existence of a unique and independent bacterial microbiota in breast tumors. We observed some similarities in breast cancer microbiota with skin; however, breast tumor microbiota was mainly enriched with Gram‐negative bacteria, serving as a primary source of lipopolysaccharide (LPS). In addition, dextran sulfate sodium (DSS) treatment in late‐stage tumor lesions increased LPS levels in the breast tissue environment. We also discovered an increased expression of S100A7 and low level of TLR4 in late‐stage tumors with or without DSS as compared to early‐stage tumor lesions. The treatment of breast cancer cells with LPS increased the expression of S100A7 in breast cancer cells *in vitro*. Furthermore, S100A7 overexpression downregulated TLR4 and upregulated RAGE expression in breast cancer cells. Analysis of human breast cancer samples also highlighted the inverse correlation between S100A7 and TLR4 expression. Overall, these findings suggest that the commensal microbiota of breast tissue may enhance breast tumor burden through a novel LPS/S100A7/TLR4/RAGE signaling axis.

## Graphical abstract

The role of commensal microbiota in breast tumorigenesis has been a research field of incomparable progress in last few years. In this study, Wilkie *et al*. discuss the functional role of breast tumor microbiota‐derived lipopolysaccharide, which enhances cancer growth through differentially regulating the S100A7/TLR4 expression. This study highlights the role of microbiota and associated signaling in the breast tumor microenvironment.

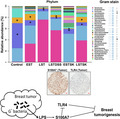

**Abbreviations**
DSS: dextran sulfate sodium
LPS: lipopolysaccharide
RAGE: receptor for advanced glycation products
TLR4: Toll‐like receptor

## 1 Introduction

Bacterial microbiota is the whole bacterial flora present in a specific organ of the human body. A decade ago, the importance of microbiota (also known as ‘the forgotten organ’) in human health started to get increased scientific attention [1]. It is known that several human organs, including skin, intestine, oral cavity, urinary tract, and the female reproductive tract, harbor a significantly higher amount of microbiota [2, 3, 4]. Increasingly in the last few years, the pathogenesis of multiple diseases, including cancer, has been associated with the human intestinal microbiota [5, 6, 7, 8, 9]. The investigations on organs that are ‘microbiota‐heavy’ or pathologically infected by bacteria suggest mainly a cancer‐promoting role of the microbiota/bacterial infection [8]. However, these studies have not yet extended well to breast cancer.

It is commonly assumed that normal and cancerous breast tissues are sterile until there is no tissue injury. However, a few reports on nonpathogenic bacterial microbiota in human breast tissues suggest that breast tissue commensal microbiota may play an important role in cancer development [10, 11, 12, 13, 14]. Antibiotic treatments that destroy commensal microbiota enhance cancer risk, especially in bacteria‐abundant organs, further justifying the role of commensal microbiota in tumorigenesis [8]. Notably, increased bacterial load in breast tumor further induces genomic instability and aggravates breast tumorigenesis [13].

Inflammation contributes to genomic instability, epigenetic changes, cytokine/chemokine and growth factor secretion, ROS generation, and RNI [15]. The inflammatory state of the tumor microenvironment is maintained by increased recruitment of various immune‐suppressive cells [16, 17]. This inflammatory milieu fuels cancer growth through the induction of several cytokines, chemokines, S100 proteins, their cognate receptors in tumor cells, and their subsequent activation of several transcription factors [18, 19]. Others and we have shown that S100 proteins including S100A7 enhance cancer growth and metastasis by creating an inflammatory tumor microenvironment by recruiting myeloid cells [20, 21]. S100A7 is a well‐known inflammatory protein that possesses antimicrobial functions [22]. Furthermore, it enhances the aggressive behavior of invasive basal‐like and ER‐negative breast cancer [23, 24, 25, 26]. Previously, we have shown that overexpression of mS100a7a15 in mouse mammary glands increases breast tumor burden and metastasis to the lungs by activating RAGE‐mediated downstream signaling pathway [25]. Phylogenetic analyses have shown that mouse ancestor mS100a7a15 is the most closely related human orthologue of S100A7 [27]. Furthermore, others and we have also shown that high expression of RAGE enhances breast tumor growth and metastasis [21, 28].

Notably, numerous studies have demonstrated the essential role of Toll‐like receptor 4 (TLR4) in anticancer immunity and breast tumor elimination by activating different immune cells, especially CD8+ T lymphocytes [29, 30]. The whole‐body deletion of TLR4 was also found to increase breast tumor growth and distant lung metastasis [31]. Furthermore, LPS has been reported to enhance the invasiveness and metastasis of breast cancer cells by activating multiple inflammatory and oncogenic signaling pathways [32, 33, 34]. However, the microbiota‐derived LPS‐mediated regulation of S100A7 and TLR4 in breast cancer is yet unexplored.

In this investigation, we sought to analyze breast tissue microbiota using an immune‐competent breast cancer mouse model and explored the role of commensal microbiota in breast cancer development. Our results have revealed for the first time that LPS‐mediated overexpression of S100A7 attenuates TLR4‐mediated effects in breast tumorigenesis.

## 2 Materials and methods

### 2.1 Cell culture and other reagents

E0.2 cells were maintained in DMEM supplemented with 10% FBS and 1% dual antibiotics. E0.2 cell line is a metastatic subclone of E0771 cell line generated in our laboratory [38]. MVT‐1 cells (derived from MMTV‐c‐Myc; MMTV‐VEGF bitransgenic mice) were obtained from Johnson [35] and were cultured as described previously [21]. The SUM159 cell line was kindly provided by S. Majumder (The Ohio State University) and cultured as described earlier [36]. The human breast carcinoma cell lines, MDA‐MB‐231 and MDA‐MB‐468, were obtained through ATCC. S100A7 overexpression or knockdown cells were generated from our previous studies [25, 37]. PrestoBlue dye (Invitrogen, Eugene, OR, USA) was used to calculate cell viability. LPS and LPS blocker (polymyxin B, PMB) were purchased from Sigma‐Aldrich. All the cell lines were routinely checked for mycoplasma contamination and verified based on cell morphology. shRNAs targeting mouse Tlr4 (Locus ID 21898) were purchased from Origene and transfected with Lipofectamine 2000 (Invitrogen, Carlsbad, CA, USA) into MVT1 cells. Breast tumor microarrays (TMAs; BR1002b) were purchased from US Biomax, Inc (Rockville, MD, USA). The clinicopathological details of TMA are available in Table S1.

### 2.2 Orthotopic breast cancer mouse model study and DSS treatment

Wild‐type C57BL/6 mice were obtained from Charles River Laboratories (Wilmington, MA, USA). At 6‐week age, 1 × 10^5^ E0.2 cells in 100 μL 50% PBS/Matrigel (Corning, MA, USA) were injected into the right mammary fat pad of the female mice as described previously [38]. For DSS treatment, after 10 days of cell injection, mice were exposed to 3% DSS in the drinking water for 7 days. On the 21^st^ day, mice were sacrificed and operations were performed using sterile techniques in a biosafety hood. All mice were housed in sterile ventilated cages in the OSU animal facility in compliance with the guidelines and protocols approved by the IACUC.

### 2.3 Microbiome study

To understand the role of resident microbial flora in the development of breast cancer, 16S‐based high‐throughput NGS metagenomics study was carried out in different biologically independent samples obtained from normal mammary glands (NMG, *N* = 2), early‐stage (EST, *N* = 3) tumors, late‐stage (LST, *N* = 4) tumors, aggressive late‐stage tumors obtained after treatment with 3% DSS in drinking water (LSTDSS, *N* = 3), and the skin from early‐stage tumor (ESTSK, *N* = 3) and late‐stage tumor (LSTSK, *N* = 4). All samples from tumor‐ and non‐tumor‐bearing mice were collected in a biosafety cabinet and were immediately snapped frozen in liquid nitrogen. The 24 frozen samples were submitted to LC Bioscience (Houston, TX, USA). Out of 24, 19 samples were then subjected to 16S‐based NGS metagenomics study after a quality control check. The specific primers designed to target the variable V3 and V4 regions of 9 variable (V1 to V9) and 10 conserved regions of 16S rDNA sequence (1542 bp) were used to generate an amplicon of about 465 bp in length. The amplified library was used for sequencing on the NovaSeq platform paired‐end reads (2 × 250 bp). We included the corresponding nape skin samples from the above‐mentioned early‐stage (ESTSK) and late‐stage (LSTSK) tumors to discern whether the microbiota of the skins and the corresponding tumors were unique from or similar to each other. All procedures were performed in a sterile biosafety hood, and spare surgical tools were placed in the same hood to serve as a negative control. All tumors were generated from the mycoplasma‐negative cell lines. We avoid those parts of tumors in late stage that had some ulcerations because ulcerations may completely modify the tumor microbiota and thus give biased outcomes.

### 2.4 Bioinformatics analysis of microbiome study

Upon the completion of NGS sequencing, the raw data were processed by merging paired‐end with overlaps, followed by quality control and chimera filtering to obtain high‐quality clean data. The later was then analyzed employing the DADA2 (Divisive Amplicon Denoising Algorithm) program to get the precision at the single‐base level of the representative sequence suitable to identify species with high accuracy and resolution [39]. The DADA2‐based analysis was subjected to denoising to construct the OUT (Operational Taxonomic Units) table and finally feature table and feature sequence, and subsequent steps of diversity analysis, species classification annotation, and differential analysis as previously reported [40]. Further analysis of the data was carried out using qiime2 software (decentralized microbiome analysis package, http://qiime2.org) [41]. Based on the feature abundance and the taxonomy annotation, the top 30 taxa were selected to plot the stacked bar chart for each sample and the cluster analysis. To determine the phenotype of the microbiome in different groups of the sample, the bugbase program was employed to predict eight major potential phenotypes including aerobic, anaerobic, facultative anaerobic, biofilm‐forming, Gram‐negative, Gram‐positive, potentially pathogenic, and stress‐tolerant [42, 43]. Finally, analysis of significant species differentiation was based on the relative abundance table of the samples. Fisher's exact test was applied to evaluate the differences between samples without biological replicates, the Mann–Whitney *U*‐test was utilized to compare the differences between two groups of samples with biological duplicates, and the Kruskal–Wallis test was applied to compare the significant differences (*P*‐values< 0.05) between groups with biological duplicates to evaluate qualitative and quantitative relative bacterial species differentiation in various tumor samples included in the study.

### 2.5 LPS (endotoxin) detection

Lipopolysaccharide in the breast tissues was detected using Pierce LAL Chromogenic Endotoxin Quantitation Kit (Thermo Scientific, Rockford, IL, USA) as per the manual. Breast tissues were homogenized, and supernatants were isolated by centrifugation before detection.

### 2.6 Western Blotting (WB), immunohistochemistry (IHC), and Immunofluorescence (IF)

S100A7 (PA5‐79947; Invitrogen), TLR4 (sc‐293072; Santa Cruz), RAGE (sc‐33662; Santa Cruz), pNF‐kB (sc‐166748; Santa Cruz), GAPDH (sc‐365062; Santa Cruz), and β‐actin (sc‐376421; Santa Cruz) antibodies were used for WB, IHC, and IF as previously described [44, 45, 46, 47]. IHC results were analyzed by using the IHC profiler imagej plugin as described earlier [48, 49]. Only high positive stained percentage of cells were considered for statistical analysis of IHC data as described previously [50].

### 2.7 Chemotaxis, wound healing, and invasion assays

Chemotactic assays were performed using transwell chambers (Costar 8 μm pore size) as described before [25, 51]. Briefly, breast cancer cell lines MVT1‐vector and MVT1‐TLR4 knockdown (KD) cells were serum‐starved for 4 h. Following serum starvation, 150 μL of 1 × 10^6^ cells·mL^−1^ in serum‐free medium (SFM) were added to top chambers and 600 μL of SFM containing murine S100A7 (100 ng·mL^−1^ mS100a7a15) was poured into the bottom chambers as described in previous study [21]. For wound healing and invasion assays, serum‐starved 231 vectors (231V) and S100A7 overexpression (S7OE) cells were treated with PBS or 100 ng·mL^−1^ LPS and analyzed for wound healing and invasive abilities as described earlier [52].

### 2.8 Quantitative Reverse Transcription–Real‐time PCR (qRT‐PCR)

RNA was isolated using TRIzol reagent (Invitrogen). Reverse transcription–PCRs were performed using RT‐PCR kits (Applied Biosystem, Carlsbad, CA, USA). The gene expression analyzed by qRT‐PCR was normalized to GAPDH using the $2^{‐\Delta \Delta C_{T}}$ method as described earlier [21, 53, 54]. The sequences of qRT‐PCR primers are given in Table S2.

### 2.9 Computational analysis and statistical analysis

Publically available datasets were analyzed for the expression of S100A7 and TLR4 using TISIDB database [55]. The Kaplan–Meier (KM) plotter was used to analyze the association between S100A7 and TLR4 expression with overall survival probability of basal subtype of breast cancer patients. Two‐sample *t*‐tests were applied if two groups were compared, and one‐way ANOVAs were used if more than two groups were compared [21]. For IHC analysis, a nonparametric Kolmogorov–Smirnov (KS) test was used to calculate the *P*‐values. For the correlation study, Spearman's rho analysis was applied. *P* < 0.05 was considered statistically significant. For all graphs, ‘*’ indicates *P* < 0.05; ‘**’ indicates *P* < 0.01; ‘***’ indicates *P* < 0.001; and ‘ns’ indicates not significant.

## 3 Results

### 3.1 Microbiota of the cancerous breast tissues is distinctly dominated by Gram‐negative bacteria

It has been reported that breast tissues can harbor different types of commensal bacteria [55, 56]; however, the association of breast tumor growth with the differential distribution of bacteria is largely unknown. Thus, in the present study, we employed an unbiased 16S NGS metagenomics approach in six different groups of samples each with multiple biological replicates to obtain comparative and quantitative changes in detailed taxonomic aspects of the microbiome.

To analyze the association of microbiota with breast tumor growth, we used an orthotopic breast cancer mouse model in which breast tumors were generated by injecting E0.2 breast cancer cells into the mammary fat pads. Mammary fat pads isolated from the non‐tumor‐bearing mice that were sham‐injected with sterile PBS instead of cancer cells were treated as normal mammary gland (NMG) samples and served as the control group. From a group of the tumor‐bearing mice, breast tumors were isolated after palpable tumor onset (< 100 mm^3^, day 10 postinjection) and were treated as an early‐stage tumor group (EST) (Fig. S1A,B). The other group of tumor‐bearing mice was left to develop late‐stage (LST) tumors (Fig. S1A,B). To determine whether the microbiome population in breast cancer is distinct from the gut‐derived leaked microbiome, a separate group of tumor‐bearing mice were treated with DSS (dextran sodium sulfate) in their drinking water to induce aggressive tumor (LSTDSS) (Fig. S1A,B).

**Fig. 1:**
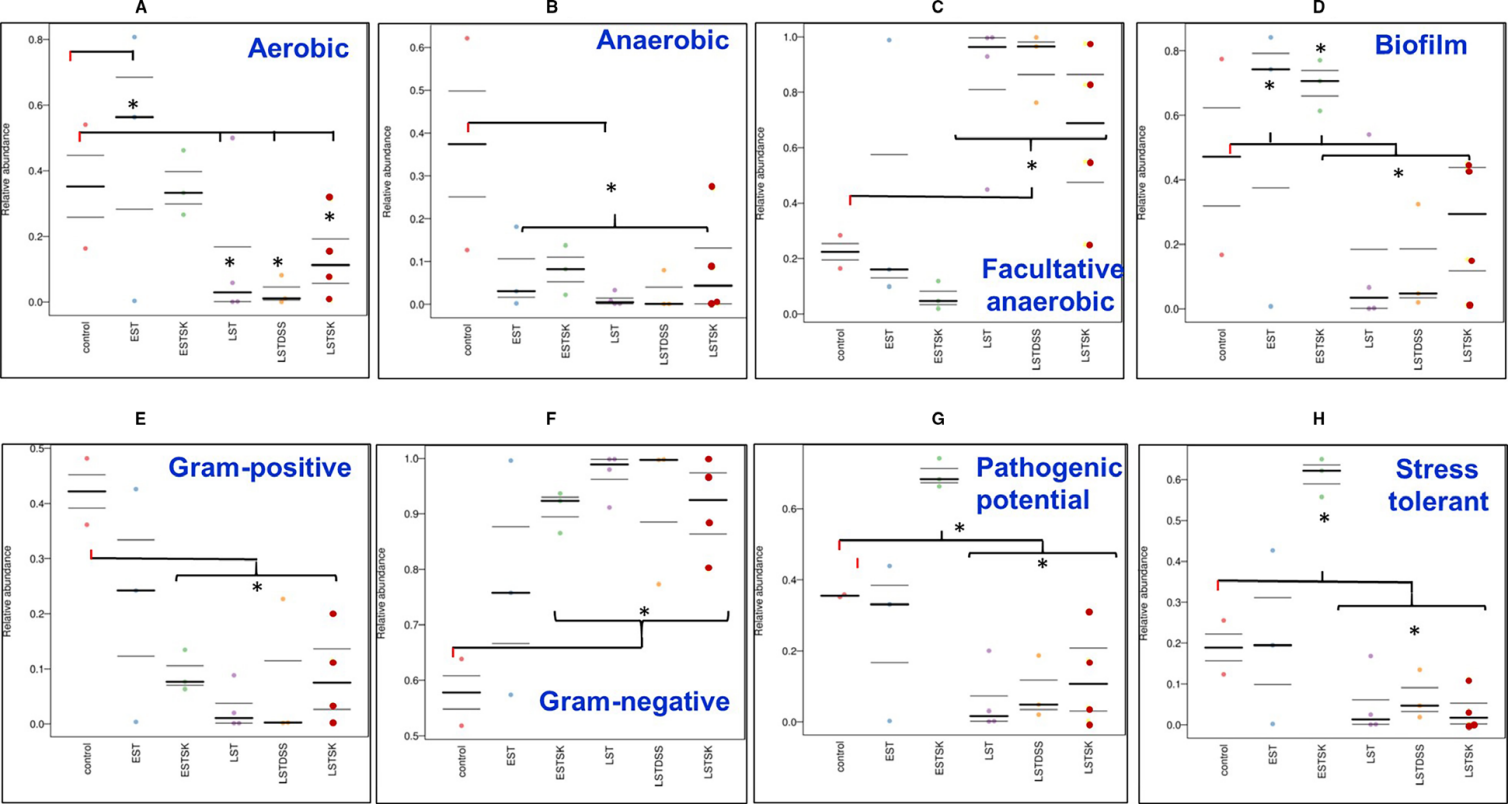
Comparative phenotypes of microbiome samples. Scatter plots showing the relative abundance of predicted phenotypes of different groups found in EST (early stage), ESTSK (early‐stage skin), LST (late stage), LSTDSS (late‐stage dextran sodium sulfate‐induced), and LSTSK (late‐stage skin) breast tumor samples. Predicted phenotypes include (A) aerobic, (B) anaerobic, (C) facultative anaerobic, (D) biofilm‐making, (E) Gram‐positive, (F) Gram‐negative, (G) microbiota that is potentially pathogenic, and (H) stress‐tolerant ability using the bugbase taxonomy prediction software. The gray horizontal line in each graph represents median values derived from the three biological replicates of each of the EST, LSTDSS, and ESTSK groups, four biological replicates of each of the LST and LSTSK groups, and two biological replicates of the control group (NMG). *indicates significant (*P* < 0.05) up or down relative abundance as compared to the control group as indicated in the figure and evaluated based on the Kruskal–Wallis and Mann–Whitney *U*‐test as described in Materials and methods.

Our results on the basic nature of microbiota in different groups as illustrated in Fig. 1 indicated that normal mammary glands harbored essentially both aerobic and anaerobic bacteria in equal abundance, but the majority of them were comprised of Gram‐positive bacteria (Fig. 1A,B,E,F). On the other hand, the microbiota of all other tumor groups was composed of Gram‐negative bacteria (Fig. 1A,B,E,F). Microbiota composition of the early‐stage tumors also differed from the late‐stage tumors; early‐stage tumors showed a high abundance of aerobic Gram‐positive bacteria as compared to late‐stage tumor having higher pathogenic potential along with biofilm‐forming and stress‐tolerant nature (Fig. 1D,G,H). The nature of microbiota of the late‐stage tumors was also essentially composed of facultative Gram‐negative bacteria (Fig. 1C).

**Fig. 2:**
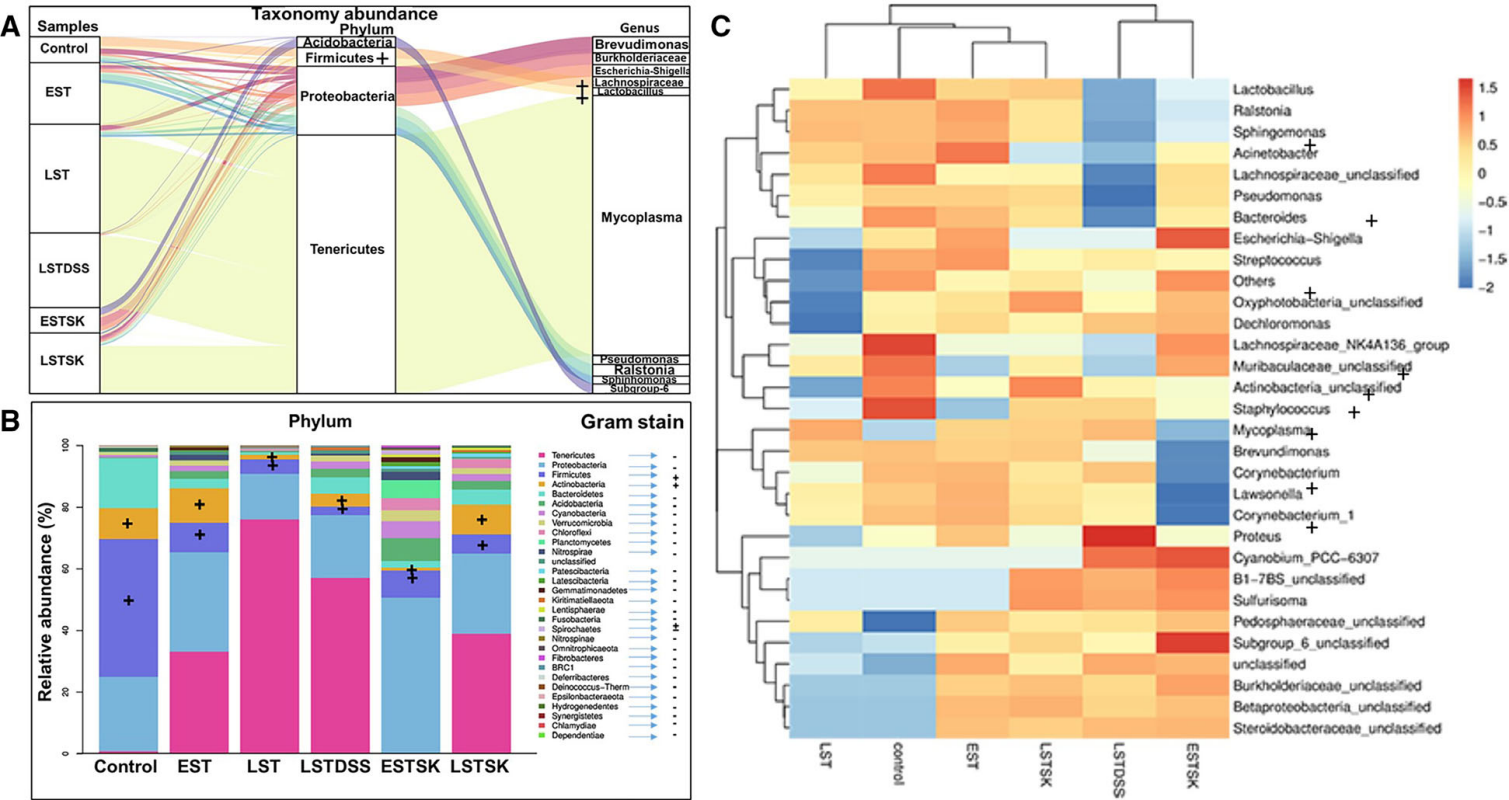
The relative abundance of bacteria at phylum level and genus level corresponding to different samples. (A) The Sankey plots showing the relative abundance of bacteria at phylum level (middle) and genus level (right) corresponding to different samples (left), visually displaying the species annotation information, corresponding relationship, and proportion of the two levels. The plot shows flow changes of the data, and the width of the plots indicates the size of the flow. (B) Average taxonomy community abundance in a different group of samples. The horizontal axis in the figure is the name of the sample, while the vertical axis represents the relative abundance of a certain classification. Different colors correspond to different phylum indicate the composition and expression of species within and between groups. According to the sample species abundance table, the 30 species with the highest abundance were selected by default for classification or functional classification. The plus (+) sign in different columns and at the right side next to the list of phylum indicates Gram‐positive organisms. (C) The heat map showing the mean value within the group is selected to represent the relative abundance of the group. Blue indicates lower abundance, and red indicates higher abundance. The right‐side list is 31 major significant genera identified in a different group (horizontal axis). The + (plus) sign indicates Gram‐positive bacteria. The rest of the genera are Gram‐negative. The gradient scale (extreme right side) from blue to red in the heat map reflects the change of abundance from low to high. The closer to blue and red indicates the lower the abundance and the higher the abundance, respectively. The results are derived from three biological replicates of each of the EST, LSTDSS, and ESTSK groups, four biological replicates of each of the LST and LSTSK groups, and two biological replicates of the control group (NMG).

When taxonomic abundance was mined, we found that Gram‐negative bacteria are composed essentially of proteobacteria encompassing *Brevundimonas*, *Burkholderiaceae, Escherichia*, *Pseudomonas*, *Ralstonia*, *Sphingomonas,* and several unclassified Subgroup_6 bacteria. The distribution of Gram‐positive Firmicutes and Actinobacteria comprising *Staphylococcus*, *Lactobacillus*, *Corynebacterium*, *Lachnospira*, *Streptococcus,* and other unclassified proteobacteria is shown in Fig. 2A–C. Additional unique features of the breast tumor microbiota are the gradual abundance of *Tenericutes* composed primarily of *M. arginini*. These cell wall‐lacking (Gram‐negative) bacteria contributed to ~ 2% (normal mammary glands), ~ 35% (early‐stage tumors), and ~ 75% (late‐stage tumors) of the total microbiota of the respective groups (Fig. 2B). In early‐stage skin samples, *Mycoplasma* species were detected in negligible quantity; however, in the late‐stage skin samples, these bacteria contributed to ~ 40% of the total microbiota.

These results on the comparative taxonomic diversity of microbiota and their relative abundance indicated that tumor microbiota was derived in part from the resident microbiota of the skin and normal mammary gland. Since the relative abundance of the Gram‐negative bacterial population in tumors was found to be significantly large, we further conducted a detailed investigation to understand the cause and result relationship between the resident bacteria‐derived LPS and the breast tumor development.

### 3.2 S100A7 expression inversely associated with TLR4 expression in breast tumor

**Fig. 3:**
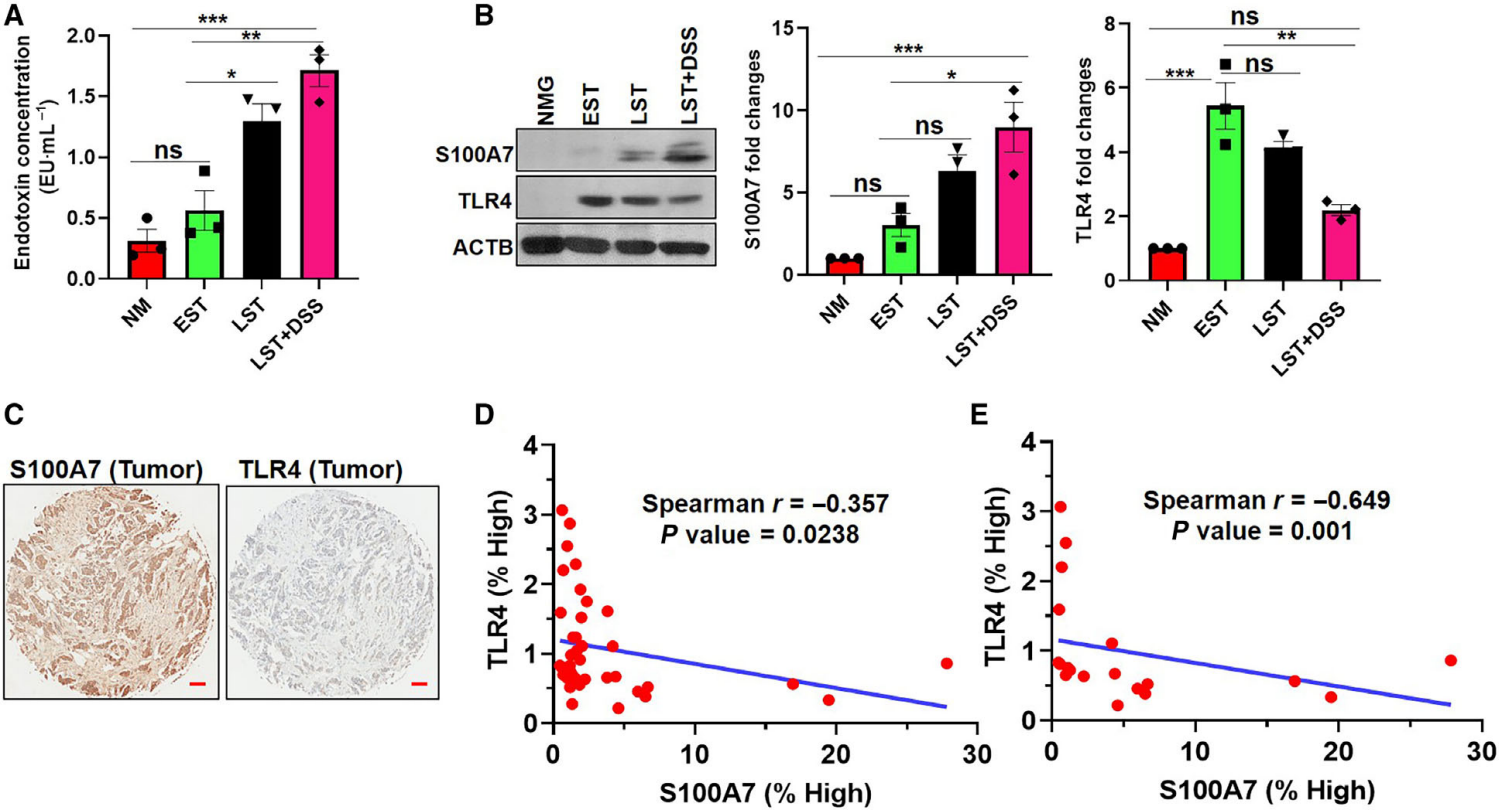
S100A7 expression inversely associated with TLR4 expression in breast tumor. (A) Bar diagram representing the level of LPS (endotoxin) in the mammary fat pad of each group. The data presented here are the mean ± SEM of three biological triplicate (*n* = 3). One‐way ANOVA (Tukey's multiple comparisons test) was used to calculate the *P*‐values. (*P < 0.05, **P < 0.01, and ***P < 0.001, ns indicates nonsignificant). (B) Expression of S100A7 and TLR4 was analyzed in tissue lysates harvested from each group by western blot. β‐Actin (ACTB) was used as a loading control. Bar diagrams represent the fold changes of S100A7 and TLR4 in three biological replicates of all the different groups. The data represented the mean ± SEM of three biological replicates (*P < 0.05, **P < 0.01, and ***P < 0.001, ns indicates nonsignificant). One‐way ANOVA (Tukey's multiple comparisons test) was used to calculate the *P*‐values. (C) Representative immunohistochemistry (IHC) images of same malignant breast tumor tissues stained with S100A7 and TLR4 antibodies. Scale bar = 300 μm. (D) Spearman’s correlation analysis of S100A7 and TLR4 protein expression in tissue microarrays (TMAs) containing malignant breast tumor tissues (*n* = 20) and their normal adjacent controls (*n* = 20). (E) Spearman’s correlation analysis of S100A7 and TLR4 protein expression only in malignant breast tumor tissues (*n* = 20). Two‐tailed *t*‐test was used to calculate the *P*‐values.

In our microbiome study, we observed that late‐stage aggressive tumors without or with DSS harbor a large number of Gram‐negative bacteria as compared to the early‐stage tumor lesion and normal mammary gland. Therefore, we investigated the level of LPS (endotoxin) to correlate with the abundance of Gram‐negative bacteria across the different samples. We found that late‐stage (LST) tumor without or with DSS revealed a significantly increased concentration of LPS as compared to the early‐stage (EST) tumor or normal mammary glands (Fig. 3A). Next, we evaluated the expression of S100A7 and TLR4 in these tissue samples and we discovered the inverse association of S100A7 with TLR4 expression in LST with or without DSS as compared to the normal mammary gland or EST samples (Fig. 3B). To corroborate the clinical association of S100A7 with TLR4 expression, we analyzed tissue microarrays (TMAs) that contain malignant breast tumors and their adjacent normal control samples. Interestingly, S100A7 showed a significant negative correlation with TLR4 expression in the same cohort of breast cancer patient samples with their adjacent normal controls (Fig. 3C,D). Furthermore, we also observed that the degree of negative correlation was increased, when we reanalyzed S100A7 and TLR4 expression only in malignant tumor tissues (Fig. 3E). Overall, increased level of LPS in the mammary tumor was found to be positively associated with S100A7 and inversely correlated with TLR4 expression. Moreover, our TMA analysis highlighted the inverse correlation of S100A7 with TLR4 expression in breast cancer patients.

### 3.3 High S100A7 expression and low TLR4 expression are poor prognostic factors in invasive breast cancer

**Fig. 4:**
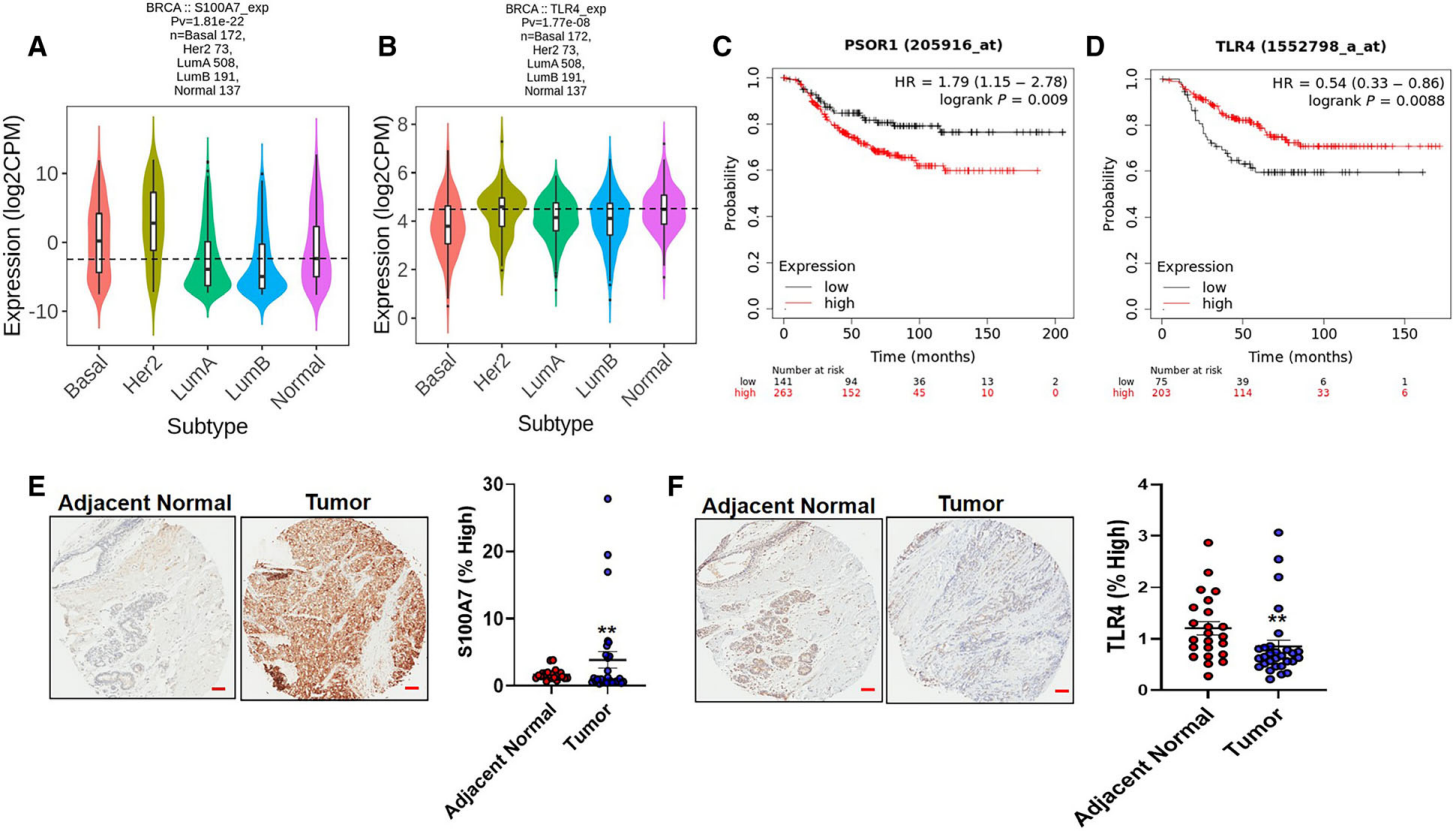
High expression of S100A7 and low level of TLR4 correlate with poor prognosis of invasive breast cancer patients. Expression of (A) S100A7 and (B) TLR4 was analyzed in normal breast tissues and different intrinsic subtypes of breast cancer by using TISIDB database. Normal (*n* = 137), LumA (*n* = 508), LumB (*n* = 191), and basal (*n* = 172). The Kaplan–Meier (KM) plotter analysis of (C) S100A7 (PSOR1) and (D) TLR4 in basal subtype of breast cancer patients. The log‐rank test was used for statistical comparison of two groups. Representative immunohistochemistry (IHC) images of (E) S100A7 and (F) TLR4 staining in malignant breast tumors (*n* = 29) and their adjacent normal controls (*n* = 23). Scale bar = 300 μm. Graphs representing the percentage (%) of high S100A7‐ or TLR4‐positive cells in malignant breast tumors (*n* = 29) and their adjacent normal controls (*n* = 23). (**P < 0.01, ***P < 0.001). A nonparametric Kolmogorov–Smirnov (KS) test was applied to calculate the *P*‐value.

To analyze the clinical significance of S100A7 and TLR4 in invasive breast cancer, we analyzed the expression of S100A7 and TLR4 by using the publically available TISIDB database [57]. Using the TISIDB database, we observed that Her2 and basal intrinsic subtypes of breast tumors showed significantly increased expression of S100A7 comparatively to normal breast tissue (Fig. 4A). Next, we also investigated the expression of TLR4 in normal and different intrinsic subtypes of breast cancer by using the same cohort of patients and normal samples. Interestingly, we discovered a decreased expression of TLR4 only in the basal intrinsic subtype of breast cancer compared with normal breast tissues (Fig. 4B). Therefore, the basal subtype is the only intrinsic molecular subtype of breast cancer, which showed an inverse pattern of expression for S100A7 and TLR4 as compared to normal samples. To analyze the patient prognosis according to the expression of S100A7 and TLR4 based on tumor‐intrinsic features, we mined the KM plotter [breast cancer] database [58]. The KM plotter analysis revealed that higher expression of the S100A7 gene had a significantly poor overall survival probability among basal intrinsic subtype of breast cancer subjects (Fig. 4C). Surprisingly, we also observed that a high level of TLR4 was significantly associated with a better survival probability of basal subtype of breast cancer patients (Fig. 4D). The basal subtype of breast cancer is a highly aggressive and metastatic molecular subtype among the different intrinsic subtypes of breast cancer [59]. In agreement with these clinical observations, we also found that malignant breast tumor tissues showed significantly increased expression of S100A7 as compared to their adjacent normal breast tissues (Fig. 4E). Finally, we analyzed the expression of TLR4 using the same TMA containing malignant and adjacent normal breast tissues. TLR4 expression was found to be significantly low in breast tumor tissues as compared to their normal adjacent breast samples (Fig. 4F). Taken together, these findings revealed that a high level of S100A7 and low expression of TLR4 could serve as poor prognostic markers for invasive breast cancer patients, especially basal or metastatic breast cancer.

### 3.4 S100A7 counteracts TLR4 in response to LPS treatment in breast cancer

**Fig. 5:**
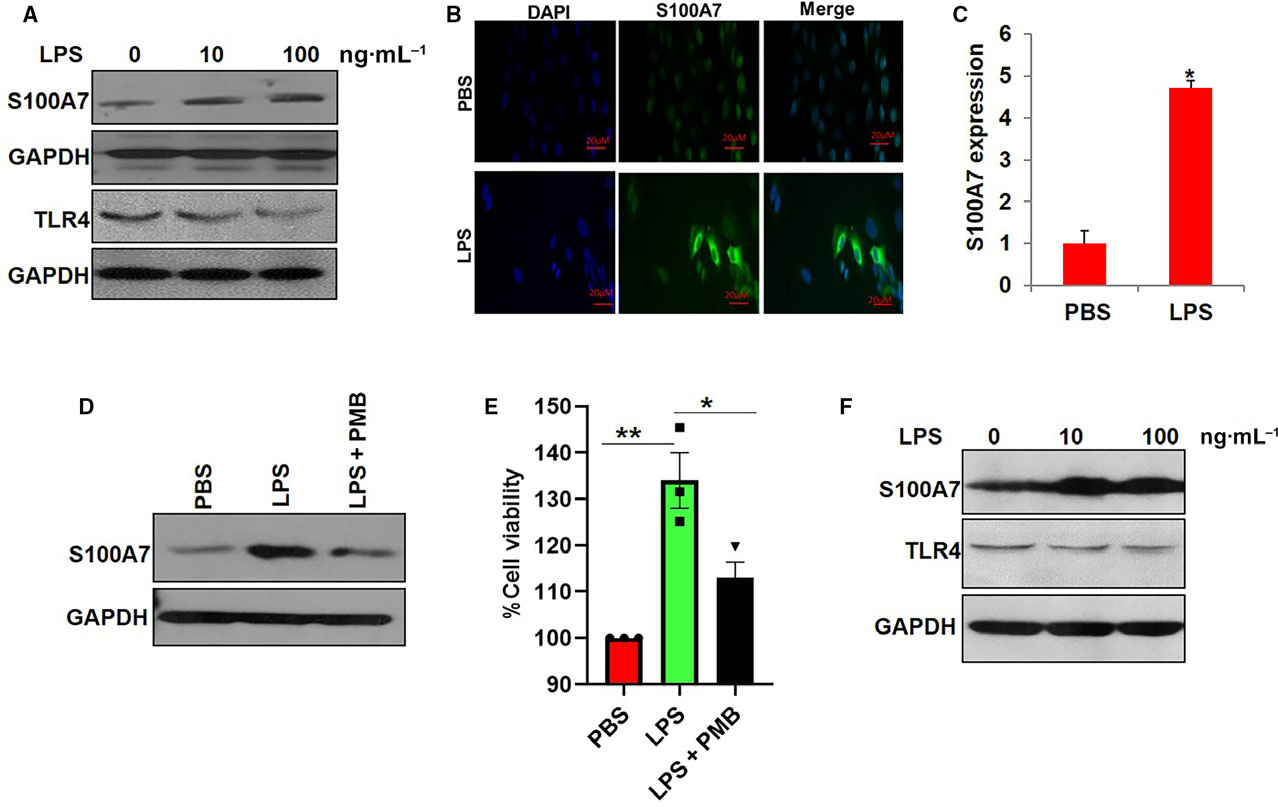
Effect of LPS treatment on the expression of S100A7 in breast cancer cells (A) SUM159 cells were treated with 10 and 100 ng·mL^−1^ of LPS or PBS (0) for 24 h, and the protein expression of S100A7 and TLR4 in SUM159 cells was analyzed by western blot analysis. GAPDH was used as a loading control. (B) Immunofluorescence (IF) analysis of S100A7 expression in PBS and LPS (100 ng·mL^−1^)‐treated SUM159 breast cancer cells. (C) The levels of S100A7 mRNA transcripts were determined in SUM159 cells after treatment with PBS or 100 ng·mL^−1^ LPS for 24 h by qRT‐PCR analysis. GAPDH was used as a loading control. The data presented here are the mean ± SEM of three biological triplicate (*n* = 3). (D) SUM159 cells after pretreatment with LPS blocker, polymyxin B (PMB), at a final concentration of 30 μg·mL^−1^ for 1 h were treated with 100 ng·mL^−1^ LPS for 24 h, cell lysates were collected, and S100A7 expression was analyzed by western blot. GAPDH was used as a loading control. (E) Cell viability analysis of SUM159 treated with PMB in the presence or absence of LPS after 24 h. The data presented here are the mean ± SEM of triplicate experiments (*n* = 3). (F) Expression of S100A7 and TLR4 was analyzed in MDA‐MB‐468 cells treated with different concentrations of LPS for 24 h. GAPDH was used as a loading control. (*P < 0.05, **P < 0.01).

**Fig. 6:**
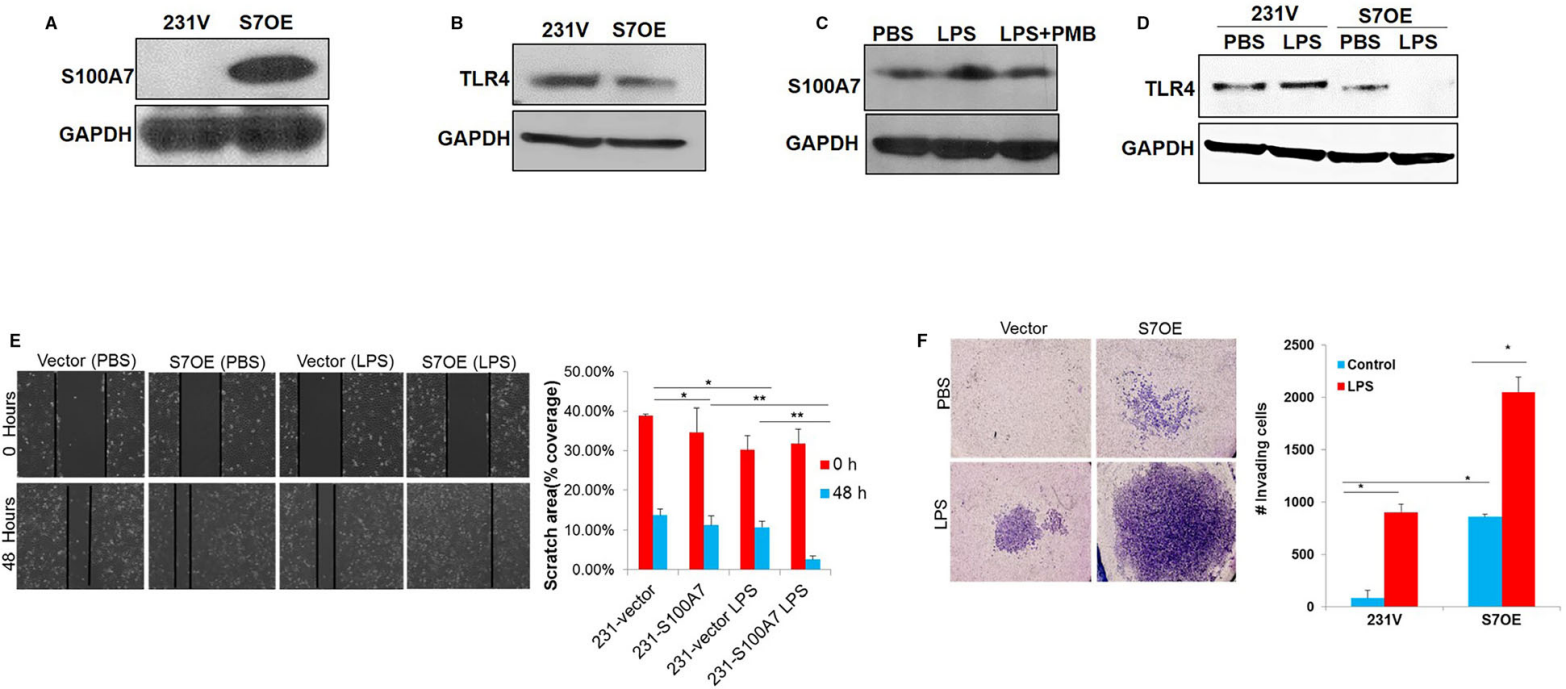
LPS‐mediated activation of S100A7 counteracts TLR4 expression in breast cancer cells. Expression of (A) S100A7 and (B) TLR4 proteins was analyzed in MDA‐MB‐231 vector (231V)‐ and S100A7‐overexpressing MDA‐MB‐231 (S7OE) breast cancer cells by western blot analysis. (C) Expression of S100A7 was analyzed in S7OE breast cancer cells treated with LPS either in the presence or in absence of PMB for 24 h. (D) Expression of TLR4 in 231V and S7OE breast cancer cells was analyzed after treatment with PBS or LPS for 24 h by western blot. GAPDH was used as a loading control. Effect of LPS on (E) wound healing and (F) migrating abilities of 231V and S7OE breast cancer cells. The data presented here are the mean ± SEM of triplicate experiments (*n* = 3). One‐way ANOVA (Tukey's multiple comparisons test) was used to calculate the *P*‐values (*P < 0.05, **P < 0.01).

As we have shown that the expression of S100A7 and LPS level increases along with an increase in tumor burden, we elucidated the effect of LPS treatment on S100A7 in breast cancer. We first evaluated the protein expression of S100A7 in LPS‐treated breast cancer cells. Our results revealed that LPS treatment increased the expression of S100A7 protein in breast cancer cells (SUM159) (Fig. 5A,B). Interestingly, treatment of SUM159 cells with different concentrations of LPS showed decreased expression of TLR4 in a dose‐dependent manner (Fig. 5A). Furthermore, LPS treatment also significantly increased the level of mRNA transcripts of the S100A7 gene in SUM159 cells (Fig. 5C). Next, we investigated the direct effect of LPS treatment in the presence or absence of LPS blocker (PMB) on S100A7 expression in SUM159 cells. We observed that LPS treatment enhanced the S100A7 expression, while treatment of PMB abrogated the LPS‐mediated activation of S100A7 in SUM159 cells (Fig. 5D). Furthermore, we found that LPS blocker significantly reduced the LPS‐induced proliferation of SUM159 breast cancer cells (Fig. 5E). We also elucidated the effect of LPS treatment on the expression of S100A7 and TLR4 in MDA‐MB‐468 cells. Interestingly, we observed that LPS treatment increased the expression of S100A7 with a reduced level of TLR4 (Fig. 5F). In addition, we investigated the effect of LPS treatment on the expression of S100A7 by using S100A7‐overexpressing MDA‐MB‐231 cells and its vector control (Fig. 6A). S100A7‐overexpressing cells revealed a decreased level of TLR4 as compared to its vector control cells (Fig. 6B). Similar to SUM159 breast cancer cells, we discovered that treatment of LPS blocker (PMB) reduced the LPS‐mediated expression of S100A7 in S100A7‐overexpressing MDA‐MB‐231 cells (Fig. 6C). Next, we evaluated the direct effect of LPS treatment on the expression of TLR4 in the presence or absence of S100A7 in breast cancer cells. Surprisingly, we found that LPS treatment decreased the expression of TLR4 only in the presence of S100A7 in MDA‐MB‐231 cells (Fig. 6D).

Further, we analyzed the effect of LPS on S100A7‐mediated wound healing and migrating abilities of S100A7‐overexpressing and vector control MDA‐MB‐231 cells. We observed that LPS treatment significantly increased the wound healing and migrating abilities of S100A7‐overexpressing cells as compared to its vector control group (Fig. 6E,F). Next, we sought to understand the functional role of the S100A7‐TLR4 axis on downstream signaling mechanism by analyzing the expression of RAGE, a well‐known receptor for S100A7. Previously, our laboratory has shown that S100A7 mediates its oncogenic effects by directly binding to RAGE in invasive breast cancer cells [21]. The treatment of MVT‐1 cells with recombinant mouse mS100a7a15 showed increased expression of RAGE in a time‐dependent manner (Fig. S2A). Interestingly, treatment of LPS on S100A7‐overexpressing MDA‐MB‐231 cells also increased the expression of RAGE in a dose‐dependent manner (Fig. S2B). Next, we downregulated the TLR4 in MVT1 cells and we discovered that downregulation of TLR4 increased the RAGE expression in these cells (Fig. S2C,D). The downregulation of TLR4 in MVT1 cells also increased the migrating ability in the presence of S100A7 in comparison with nontargeting shRNA control cells (Fig. S2E). However, we did not observe any significant change in migrating ability of MVT1 cells after TLR downregulation in the PBS‐treated group. Finally, the expression of TLR4 and RAGE was also analyzed in S100A7‐downregulated MDA‐MB‐468 cells. We observed that S100A7 downregulation increased the expression of TLR4 that in turn leads to reduced expression of RAGE (Fig. S2F). We have shown that S100A7/RAGE signaling mediates its oncogenic effect by activating the NF‐κB in breast cancer cells [21, 37]. Moreover, it has been reported that Gram‐negative bacteria such as Mycoplasma activate the NF‐κB and Ras signaling to initiate the transformation of normal fibroblasts [60]. We found that S100A7 downregulation decreased the phosphorylation of NF‐κB in MDA‐MB‐468 cells (Fig. S2F). In summary, our results indicate that S100A7 counteracts the expression of TLR4 in response to LPS treatment, which in turn activates the RAGE‐mediated downstream signaling in breast cancer cells.

## 4 Discussion

Several reports have indicated that bacteria are present in the skin‐protected tissues, such as atherosclerotic aortic plaques [61, 62, 63] and breast tissues [10, 11]. In this study, we have demonstrated that a unique commensal microbiota exists in breast tumors. In particular, we have revealed that the commensal microbiota at the late‐stage tumors is a unique and independent community in comparison with that of the normal mammary glands and early‐stage tumors. These unique features include the following: (a) majority of the bacteria belonged to facultative Gram‐negative bacteria; and (b) the dominant phylum is *Tenericutes*. The first unique feature is in agreement with a previous report on microbiota in surgically isolated human breast tissues, in which ~ 63% of the detected species was Gram‐negative [10]. The second unique feature corroborates with two other research studies of human breast microbiota, where Gram‐negative bacteria were shown as the dominant microbiota in breast tissues [10, 11, 64]. It is reasonable to conceptualize that the unique tissue content and structure in the breast are likely responsible for the prevalence of Gram‐negative bacteria. The distinct set of breast cancer microbiota akin to skin microbiota provides strong evidence of the environmentally adapted special composition of the bacteria. In addition, it rules out the possibility of contamination from other body parts during surgical procedures. The observed high similarities of the microbiota in late‐stage tumors to that of the skin may be explained with the following underlying mechanisms. The original breast cancer microbiota at tumor onset may disappear as the tumor grows as reported earlier [11]. On the other hand, at the later stage, skin bacteria may be able to invade the tumor environment through ulcerative wounds and/or overstretched skin over cancer lesions.

One of the conspicuous natures of the microbiota of the late‐stage tumors in the present study was the gradual abundance of mycoplasma species, especially *M. arginini*. The later has been known as mouse normal flora [65]. *M. arginii* has been shown to suppress p53 function and constitutively activate NF‐kB signaling, a common feature of tumor cells, which in turn contribute to their unconstrained growth [60, 66]. *M. arginini*‐induced mouse B‐cell activation requires accessory factors for cancer [67]. Whether LPS or certain metabolic products of Gram‐negative bacteria serve as accessory factors is presently unknown. Unlike LPS, membrane lipoproteins of mycoplasma activate TLR2 and TLR6 and their effect is synergized with LPS in triggering TNF‐α production by macrophages from LPS‐responsive mice. It is highly likely that in breast tumor development, mycoplasma may cooperatively interact with the S100A7/LPS signaling axis [68], although the precise mechanism and nature of this predicted interaction remain unclear.

The role of LPS in intestinal dysfunction through a complex intercellular inflammatory crosstalk has been studied in multiple animal inflammation models employing chemical, genetic, and immunological treatment [69, 70, 71]. However, such models are biased toward one individual uncoupled factor as its possible role in spatiotemporal contexts. Hence, we preferred to employ the DSS‐induced colitis model [72], which mimics the compromised intestinal barrier integrity (atrophic villous blunting without inducing epithelial cytotoxicity or epithelial cell viability) observed during inflammatory bowel disease (IBD). A recent study has shown that in the DSS‐induced colitis model, intestinal dysfunction is primarily caused by an increased abundance of Gram‐negative bacteria and infiltrated LPS from the leaky gut and its interaction with PBMC [73]. In the present study, we also found that the abundance of Gram‐negative bacterial populations increased substantially in LST and LSTDSS tumors as compared to control skin samples or EST breast tumors. In both cases, we observed significantly increased amounts of LPS as compared to control samples. We surmise that aggressive breast tumor development observed in the DSS‐induced colitis model is likely due to the infiltrated LPS of increased Gram‐negative bacteria from the leaky gut and LPS‐modulated tumor development. While an increased abundance of Gram‐negative bacterial population was observed in both LST and LSTDSS breast tumors, there was no observed significant difference in abundance between them. However, we did observe modulated genus population. In LSTDSS breast tumors, *Lachnospiraceae* was downregulated but cyanobacterium, *Betaproteobacteria*, *Burkholderiaceae*, *Dechloromonas*, *Sulfurisoma*, *Steroidobacteriaceae,* and *Muribaculaceae* population increased substantially compared with the LST tumors. A similar population was also seen in skin samples of the late‐stage tumor (LSTSK). Whether individual Gram‐negative bacteria‐derived LPS contributes differentially to the aggression of breast tumor development is presently unknown and is also technically challenging as the abundance of the local microbiota at individual genus levels is continuously changing and modulated based on the spatiotemporal microenvironment.

S100A7 is a microbicidal protein, and previous reports from our laboratory have established its role in breast cancer progression and metastasis [21, 25]. In particular, our results have elucidated how the overexpression of S100A7 induces hyperplasia in the mammary gland and recruits tumor‐associated macrophages in the S100A7‐overexpressing bitransgenic mouse model [21]. To our knowledge, the signaling between S100A7 and TLR4 in invasive breast cancer in the presence of microbiota‐derived LPS has not been established. This is the first study, which reports that LPS increases S100A7 expression in breast cancer cells, which in turn attenuates the expression of TLR4. Furthermore, we have also shown that expression of S100A7 is inversely correlated with TLR4 expression in breast cancer patients, especially in basal intrinsic subtype of breast cancer. Although few studies have reported that TLR4 enhances breast tumor growth, it has also been shown that TLR4 plays a TP53‐dependent dual role in regulating breast cancer cell growth [74, 75]. Interestingly, whole‐body deletion of TLR4 enhances the tumor growth and distant metastasis in TLR4 knockout transgenic breast cancer mouse model [31]. In the present study, we have elucidated that S100A7 can determine the fate of TLR4 in breast cancer cells, and the exogenous treatment of LPS/S100A7 or downregulation of TLR4 increases the expression of RAGE, thus confirming our previous report revealing an essential role of S100A7‐RAGE signaling in the enhancement of breast cancer growth and metastasis, especially in triple‐negative breast cancer [21]. Altogether, our study reports a novel role of LPS in driving S100A7 expression for modulating the expression of TLR4 and RAGE, which demands further exploration of this regulatory axis for drug intervention of invasive breast cancers.

## 5 Conclusion

In conclusion, our study has revealed the following key points. (a) Breast cancer commensal microbiota is distinctly composed of Gram‐negative bacteria that stands out as a sole source of LPS. (b) LPS increases S100A7 expression in breast cancer cells that regulate TLR4 and RAGE expression and modulate breast tumorigenesis. This study calls for oncologist’s attention to the previously neglected breast cancer commensal microbiota and highlights the role of bacterial components and its associated LPS/S100A7/TLR4 signaling cascade in the breast tumor microenvironment (Fig. S3).

## Conflict of interest

The authors declare no conflict of interest.

## Author contributions

SM and RKG conceived and supervised the project. TW, SM, and VP wrote the manuscript. TW, AKV, SM, HZ, MC, and SK performed most of the experiments and analyzed the data with assistance from RKG, VP, and DKA.

## Supporting information


**Fig. S1.** Analysis of breast tumor burden in E0.2 cells injected orthotopic breast cancer mouse model. Bar diagrams showing the (A) Tumor volume (mm^3^) and (B) tumor weight (gm) of EST, LST, and LST with DSS treated experimental groups. The data presented here is the mean ± SEM of triplicate experiments (** P < 0.01).

**Fig. S2.** Effect of TLR4 knockdown on RAGE expression and breast cancer cell migration. (A) MVT1 cells were treated with murine S100A7 recombinant proteins (100ng/ml) at different time points (minutes) and were analyzed for expression of RAGE by western blot. (B) Expression of RAGE in S7OE MDA‐MB‐231 cells treated with different concentrations of LPS for 24 hrs. (C & D) Expression of TLR4 and RAGE protein in MVT1 vector control (VC) and TLR4 knockdown MVT1 (TLR4‐KD) cells as analyzed by western blot. GAPDH was used as the loading control. (E) Effect of murine S100A7 recombinant proteins (100ng/ml) on migrating abilities of VC and TLR4‐KD MVT1 cells. One‐way ANOVA was used to calculate the p values. The data are mean ± SEM of triplicate experiments (*P < 0.05, *** P < 0.001). (F) Expression of S100A7, TLR4, RAGE, and pNF‐κB was analyzed in MDA‐MB‐468 vector control (Vector) and S100A7 downregulated (S7KD) cells were analyzed by western blot. β‐actin (ACTB) was used as a loading control.

**Fig. S3.** Schematic diagram showing the role of gram‐negative bacteria‐derived LPS in modulating the S100A7/TLR4 signaling in breast tumorigenesis.

**Table S1.** Pathological details of breast cancer tissue microarrays used in IHC.
**Table S2.** Sequence of primers used in qRT‐PCR.

## Data Availability

All data analyzed or generated in this study are included in this published article and its supplementary files.
