# Supplementary figures and images for: Lipopolysaccharide from the commensal microbiota of the breast enhances cancer growth: role of S100A7 and TLR4
Source: Mol Oncol. 2021 Nov 16;16(7):1508–22. doi: 10.1002/1878-0261.12975 (PMC8978520; doi:10.1002/1878-0261.12975)

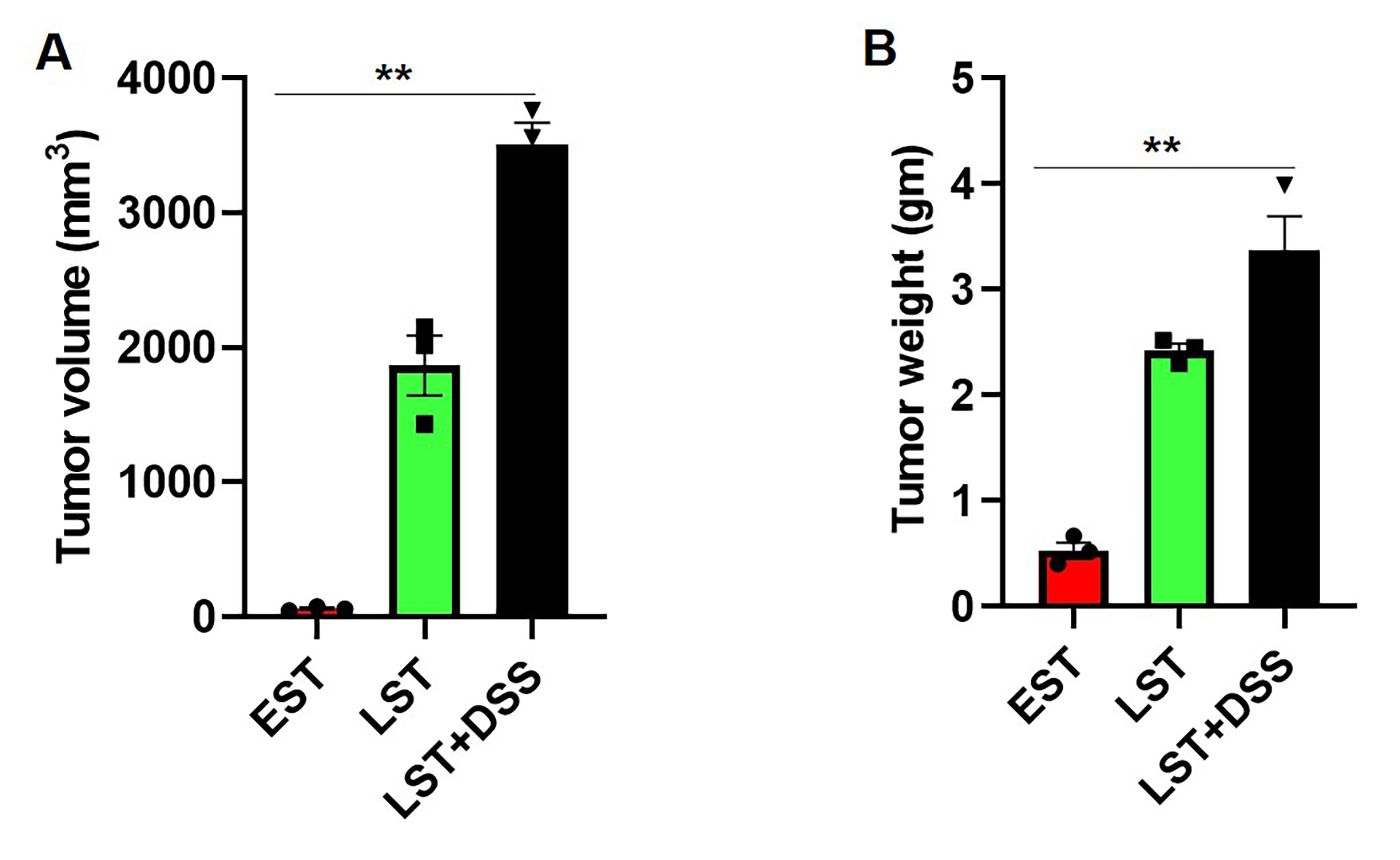

Supplement: Supplementary file 1 — Fig. S1. Analysis of breast tumor burden in E0.2 cells injected orthotopic breast cancer mouse model. Bar diagrams showing the (A) Tumor volume (mm3) and (B) tumor weight (gm) of EST, LST, and LST with DSS treated experimental groups. The data presented here is the mean ± SEM of triplicate experiments (** P < 0.01). [file MOL2-16-1508-s001.jpg]

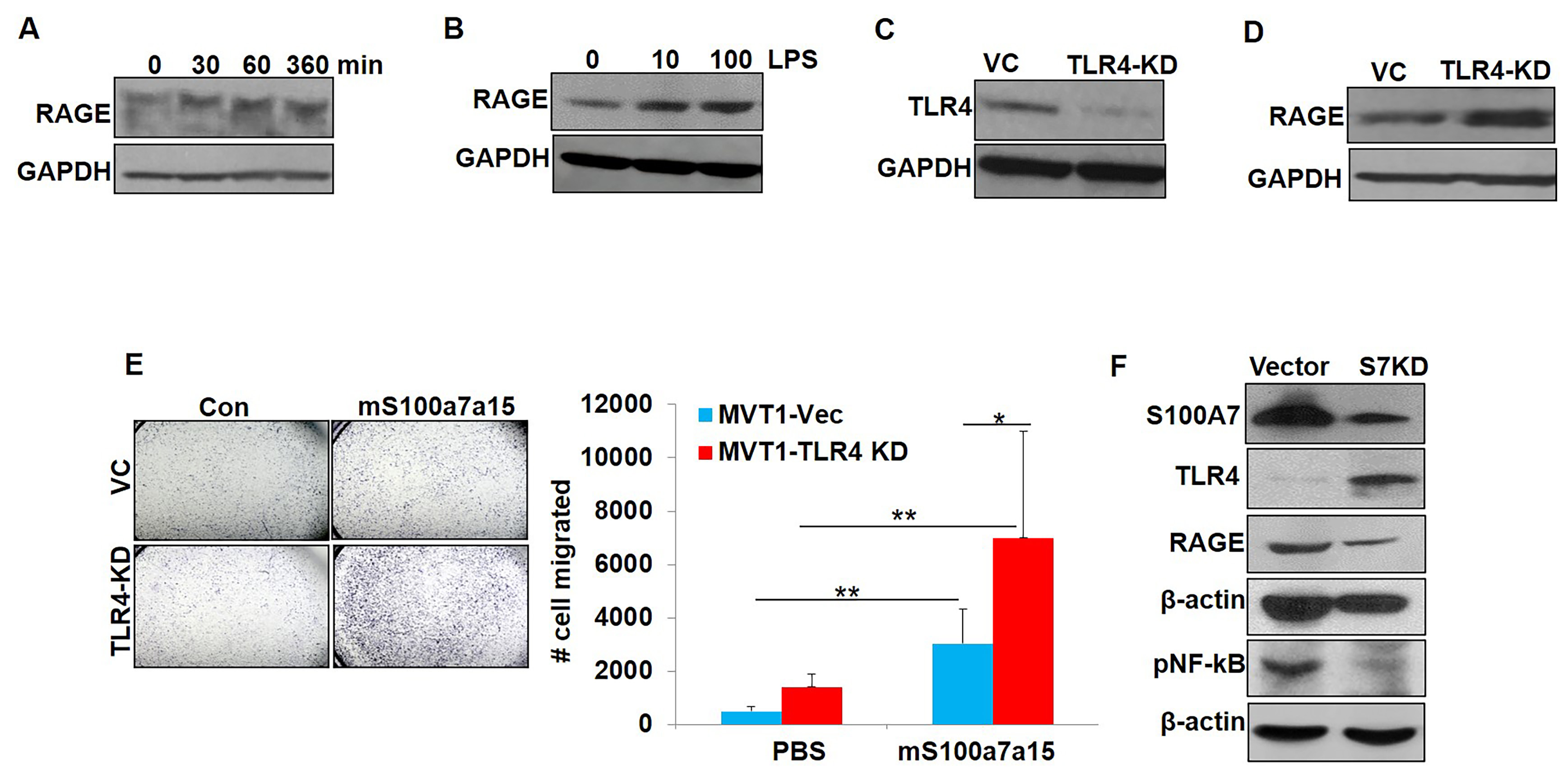

Supplement: Supplementary file 2 — Fig. S2. Effect of TLR4 knockdown on RAGE expression and breast cancer cell migration. (A) MVT1 cells were treated with murine S100A7 recombinant proteins (100ng/ml) at different time points (minutes) and were analyzed for expression of RAGE by western blot. (B) Expression of RAGE in S7OE MDA‐MB‐231 cells treated with different concentrations of LPS for 24 hrs. (C & D) Expression of TLR4 and RAGE protein in MVT1 vector control (VC) and TLR4 knockdown MVT1 (TLR4‐KD) cells as analyzed by western blot. GAPDH was used as the loading control. (E) Effect of murine S100A7 recombinant proteins (100ng/ml) on migrating abilities of VC and TLR4‐KD MVT1 cells. One‐way ANOVA was used to calculate the p values. The data are mean ± SEM of triplicate experiments (*P < 0.05, *** P < 0.001). (F) Expression of S100A7, TLR4, RAGE, and pNF‐κB was analyzed in MDA‐MB‐468 vector control (Vector) and S100A7 downregulated (S7KD) cells were analyzed by western blot. β‐actin (ACTB) was used as a loading control. [file MOL2-16-1508-s002.jpg]

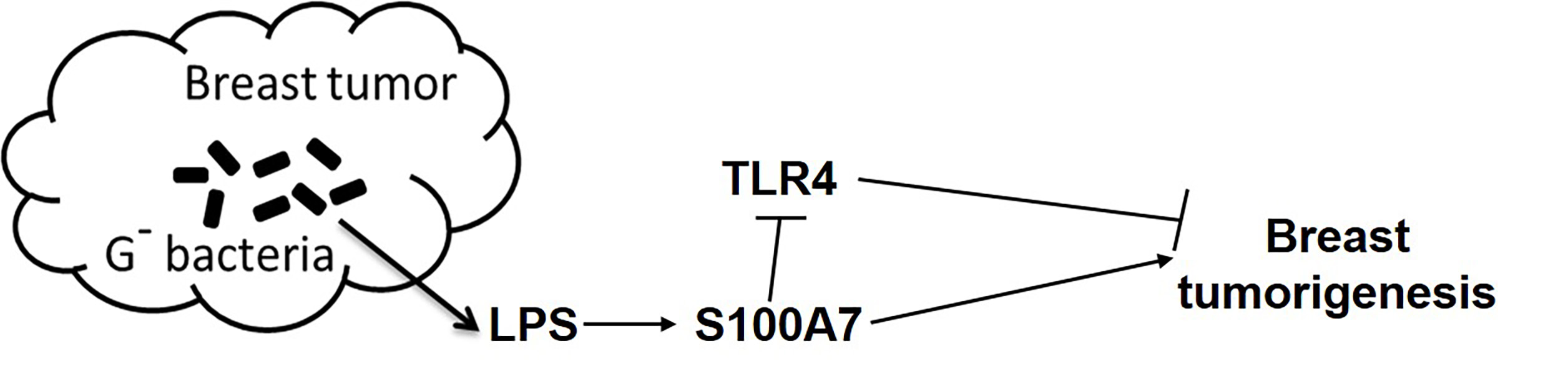

Supplement: Supplementary file 3 — Fig. S3. Schematic diagram showing the role of gram‐negative bacteria‐derived LPS in modulating the S100A7/TLR4 signaling in breast tumorigenesis. [file MOL2-16-1508-s004.jpg]
