# Supplementary material for: Lipopolysaccharide from the commensal microbiota of the breast enhances cancer growth: role of S100A7 and TLR4
Source: Mol Oncol. 2021 Nov 16;16(7):1508–22. doi: 10.1002/1878-0261.12975 (PMC8978520; doi:10.1002/1878-0261.12975)
Supplement: Supplementary file 4 — Table S1. Pathological details of breast cancer tissue microarrays used in IHC. Table S2. Sequence of primers used in qRT‐PCR. [file MOL2-16-1508-s003.docx]

**Supplementary Table 1: Pathological details of breast cancer tissue microarrays used in IHC.**

| **Samples details** | **Number (n)** |
| --- | --- |
| Normal adjacent tissue | 23 |
| Malignant breast tumor tissues | 29 |
| Grade 1 08  Grade 2 13  Grade 3 08 | |
| Stage IIA 18  Stage IIB 07  Stage IIIB 04 | |
| T2N0M0 18  T2NIM0 06  T3N0M0 01  T4bN0M0 01  T4N0M0 03 | |

**Supplementary Table 2: Sequence of primers used in qRT-PCR.**

| **Gene names** | **Forward primer (5’-3’)** | **Reverse primer (5’-3’)** |
| --- | --- | --- |
| S100A7 (Human) | ccaaacacacacatctcactca | tcagcttgagtgttgctcatc |
| GAPDH (Human) | agccacatcgctcagacac | gcccaatacgaccaaatcc |
